# Supplementary figures and images for: Dynamics of X chromosome hyper-expression and inactivation in male tissues during stick insect development
Source: PLoS Genet. 2025 Mar 10;21(3):e1011615. doi: 10.1371/journal.pgen.1011615 (PMC11957559; doi:10.1371/journal.pgen.1011615)

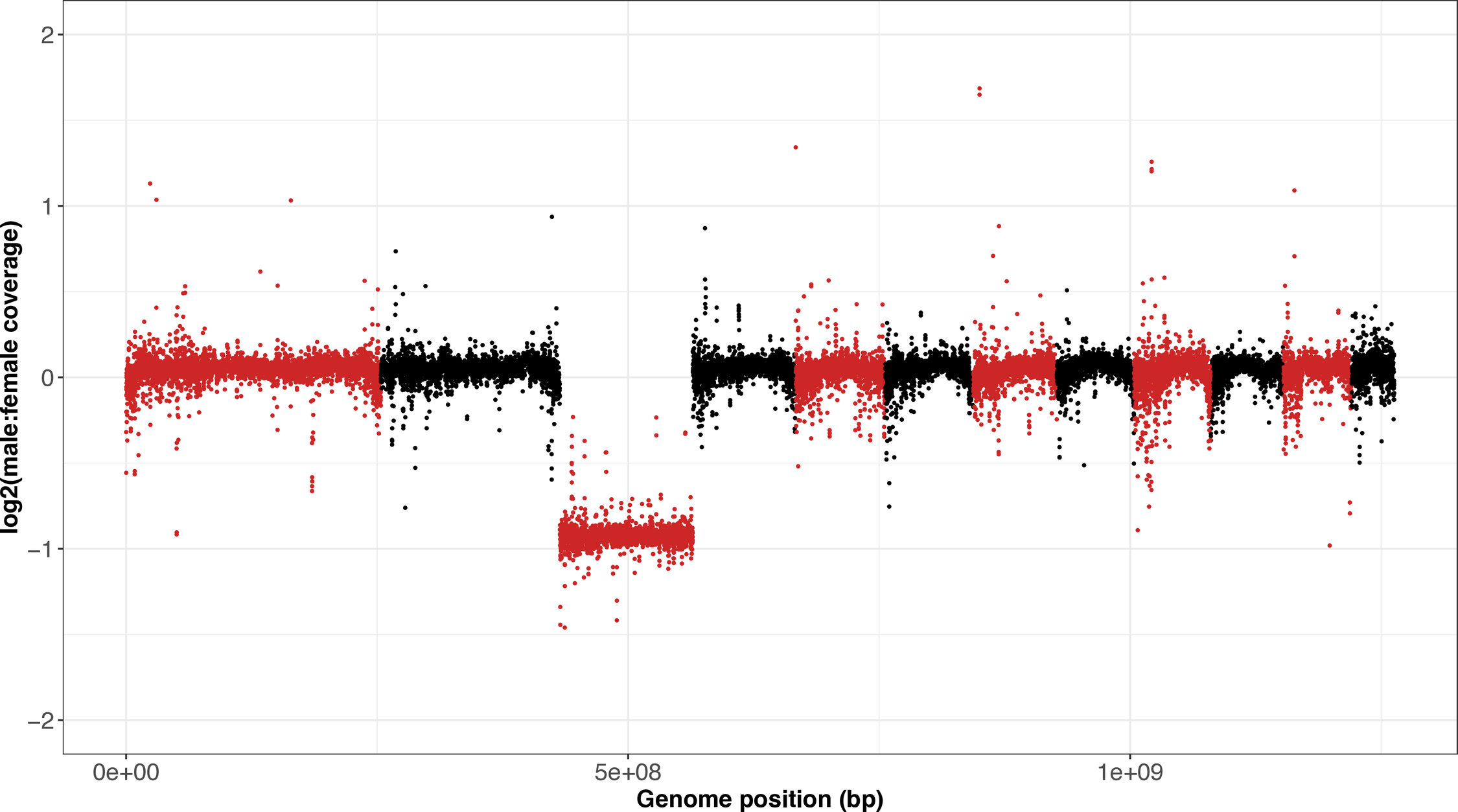

Supplement: S1 Fig — The plot shows the log2 ratio of male to female coverage of 100 kb sliding windows across the genome. Alternated colours designate different chromosomes, with chromosome 3 showing a much lower overall male to female coverage ratio. (TIF) [file pgen.1011615.s006.tif]

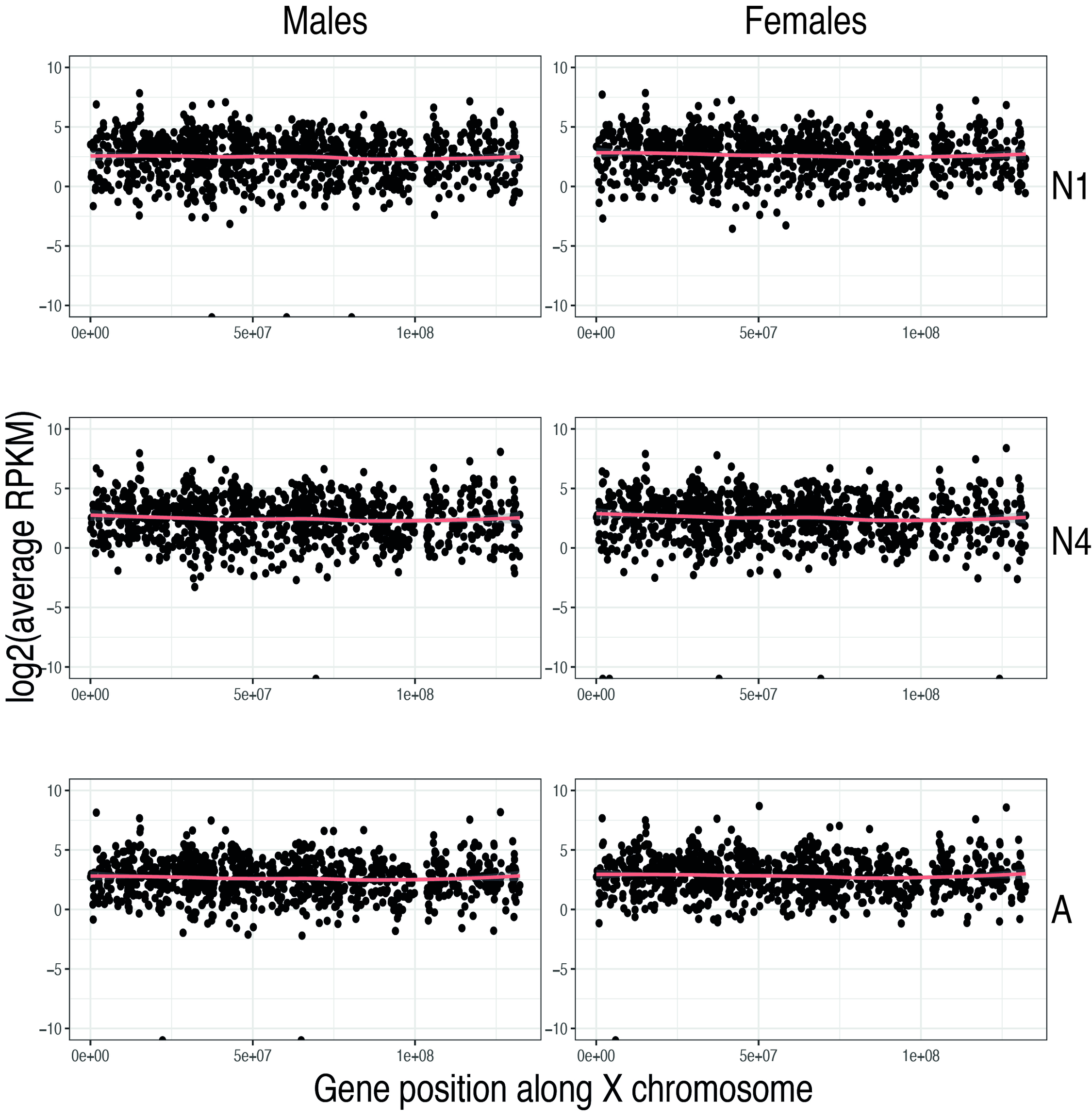

Supplement: S2 Fig — The line in each panel represents a loess (Locally Estimated Scatterplot Smoothing) smoothed curve, a non- parametric regression method that fits localized linear regressions to subsets of the data, highlighting overall expression patterns along chromosome. (TIF) [file pgen.1011615.s007.tif]

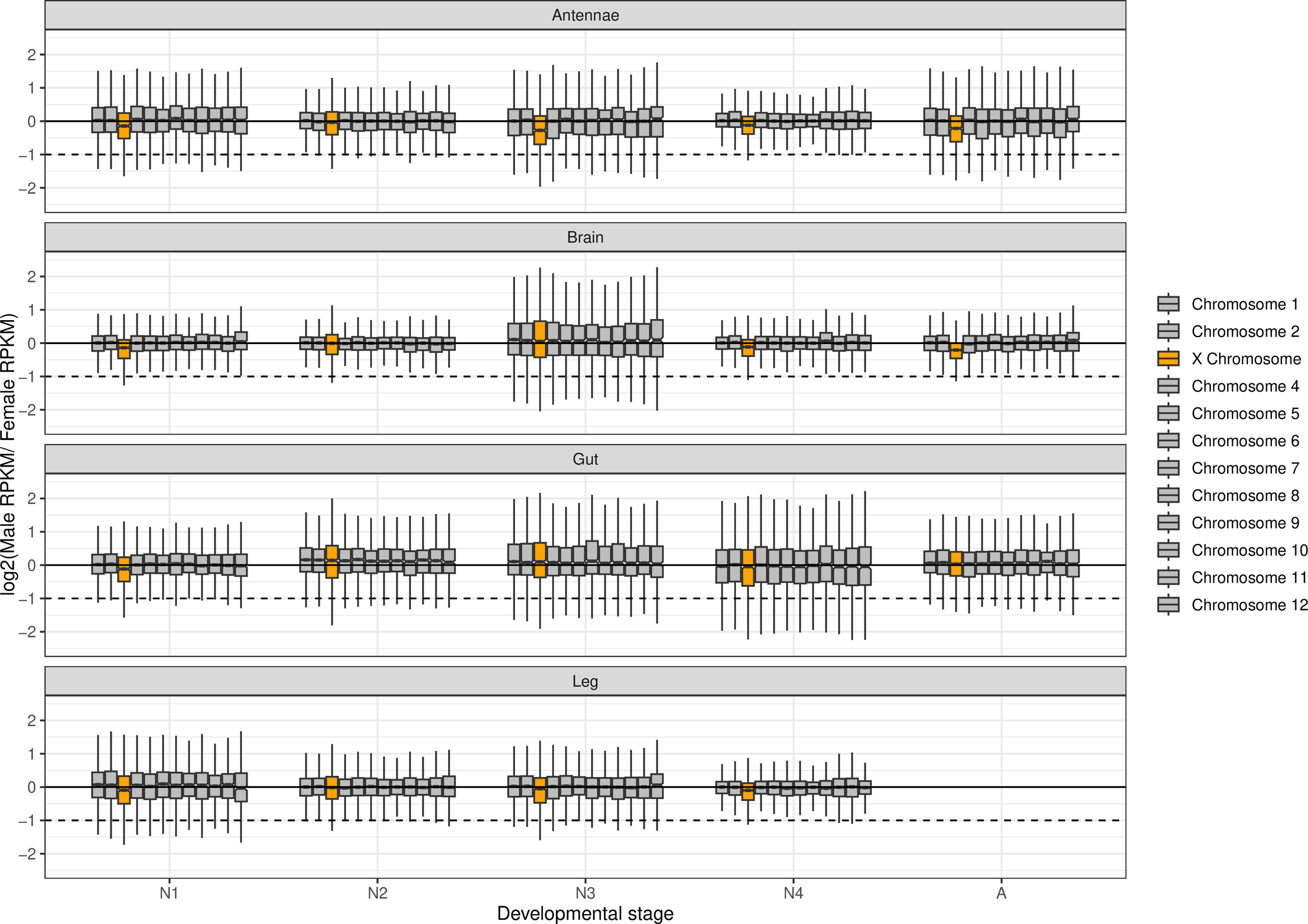

Supplement: S3 Fig — Chromosome 3, represented in orange, corresponds to the X chromosome, while autosomes are depicted in gray. The panels, arranged from top to bottom, showcase the Log2 ratio in different somatic tissues: antenna, brain, guts, and legs. Boxplots depict the median, the lower and upper quartiles, while the whiskers represent the minimum and maximum values, within 1.5x the interquartile range. (TIF) [file pgen.1011615.s008.tif]

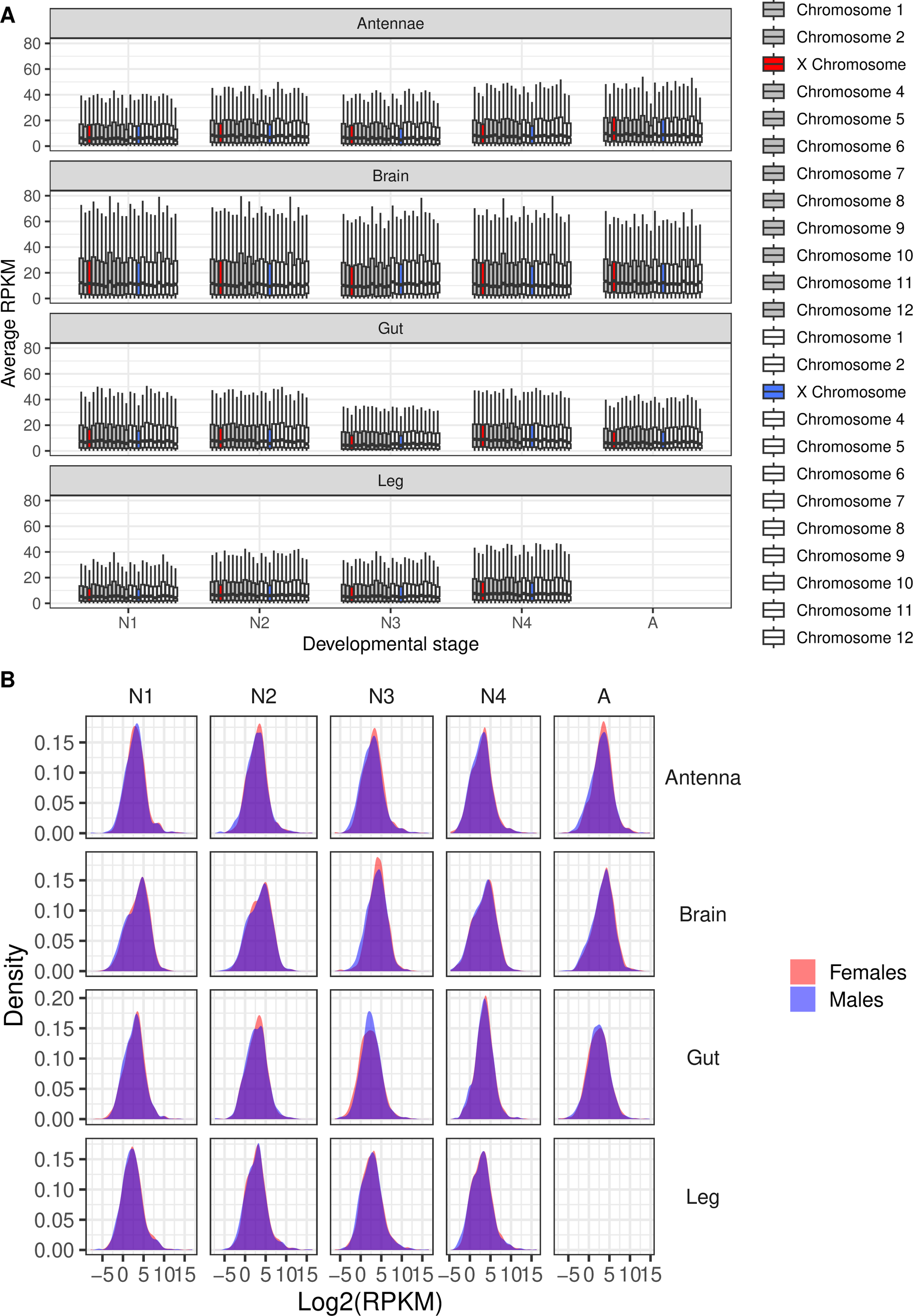

Supplement: S4 Fig — The panels, from top to bottom, showcase the Average RPKM in different somatic tissues: antenna, brain, guts, and legs. Boxplots depict the median, the lower and upper quartiles, while the whiskers represent the minimum and maximum values, within 1.5x the interquartile range. B) Distribution of expression levels for X-linked genes in males (blue) and females (red) (overlapping ranges are indicated in purple) across developmental stages and somatic tissues. (TIF) [file pgen.1011615.s009.tif]

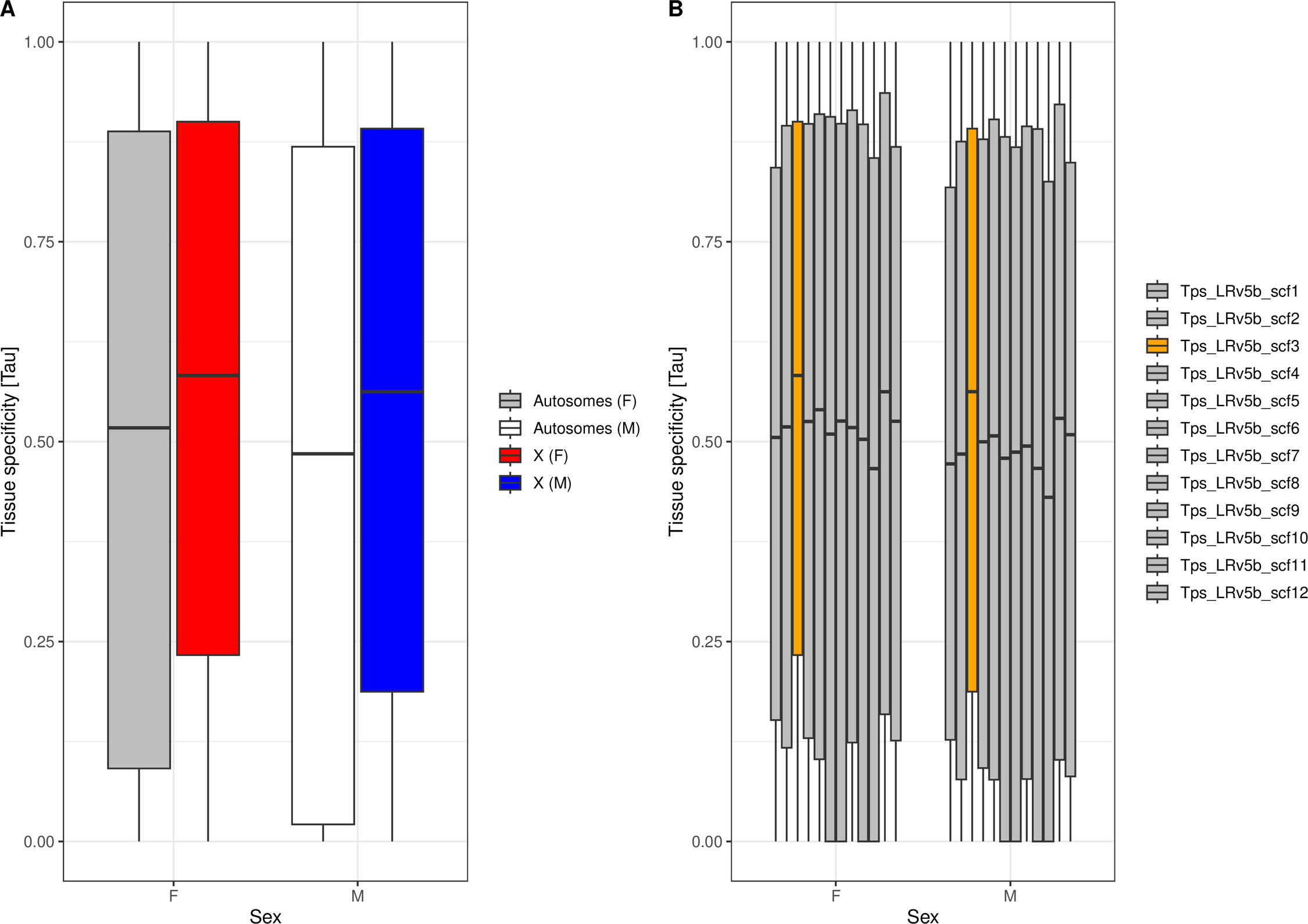

Supplement: S5 Fig — Because genes on the X are often testes or ovaries specific, we here repeated the analysis presented in the main text based on four tissues (three somatic tissues and reproductive tracts) with the three somatic tissues only, and X tissue specificity remained higher than autosomes Wilcoxon test, padj (females)= 6.7e-13, padj (males)= 2.4e-16 B) Tissue specificity in females (F) and males (M) across scaffolds (see S5 Table), based on three somatic tissues. Scaffold 3, represented in orange, corresponds to the X chromosome, while other scaffolds are depicted in gray. Boxplots depict the median, the lower and upper quartiles, while the whiskers represent the minimum and maximum values, within 1.5x the interquartile range. (TIF) [file pgen.1011615.s010.tif]

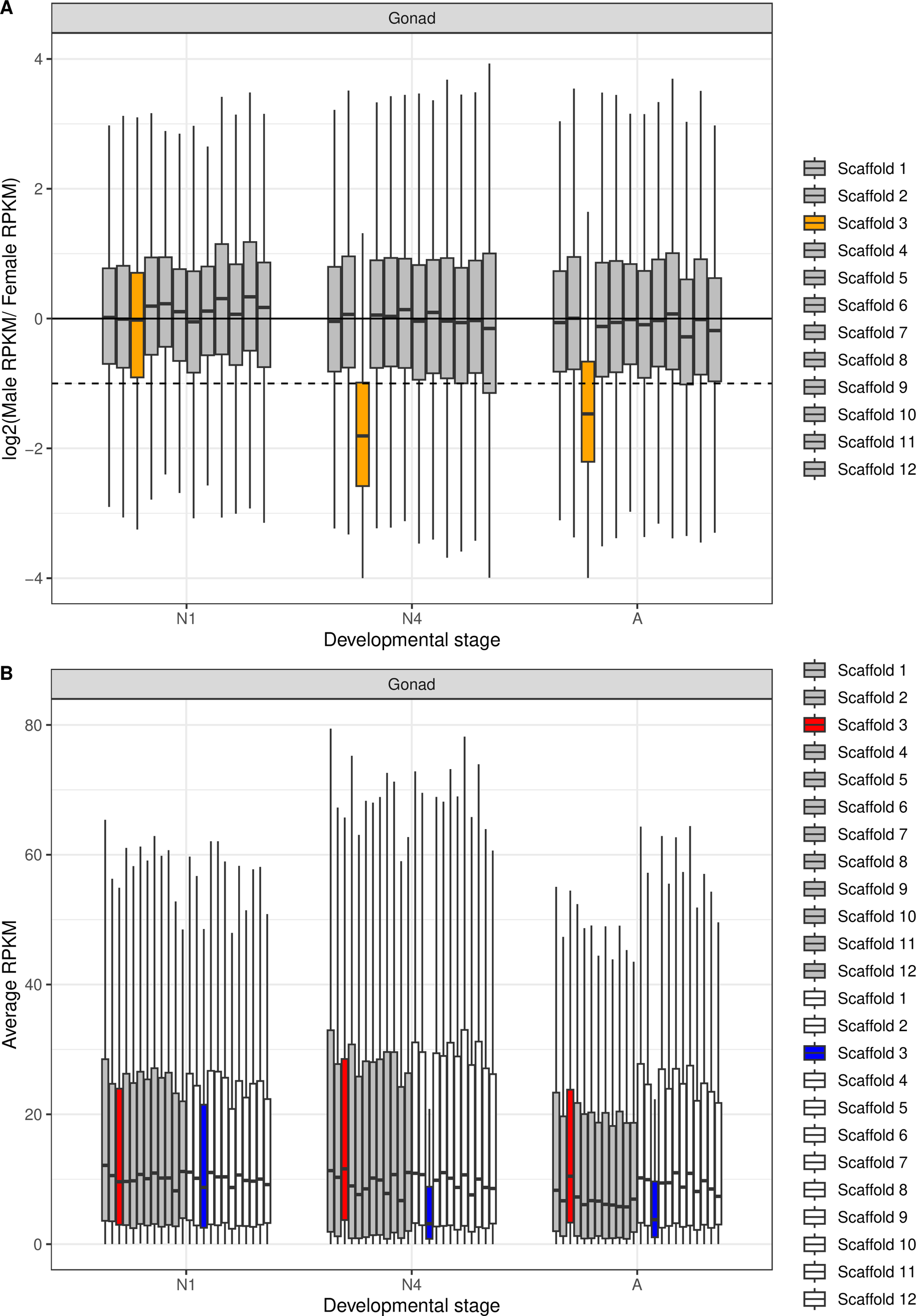

Supplement: S6 Fig — Chromosome three, represented in orange, corresponds to the X chromosome, while other chromosomes are depicted in gray. B) Average RPKM expression levels at three developmental stages (N1, N4, and adult) in the reproductive tract separated for different chromosomes in females (grey) and in males (white) boxes, chromosome three corresponds to the X and is depicted in red (females) and blue (males). Boxplots depict the median, the lower and upper quartiles, while the whiskers represent the minimum and maximum values, within 1.5x the interquartile range. (TIF) [file pgen.1011615.s011.tif]

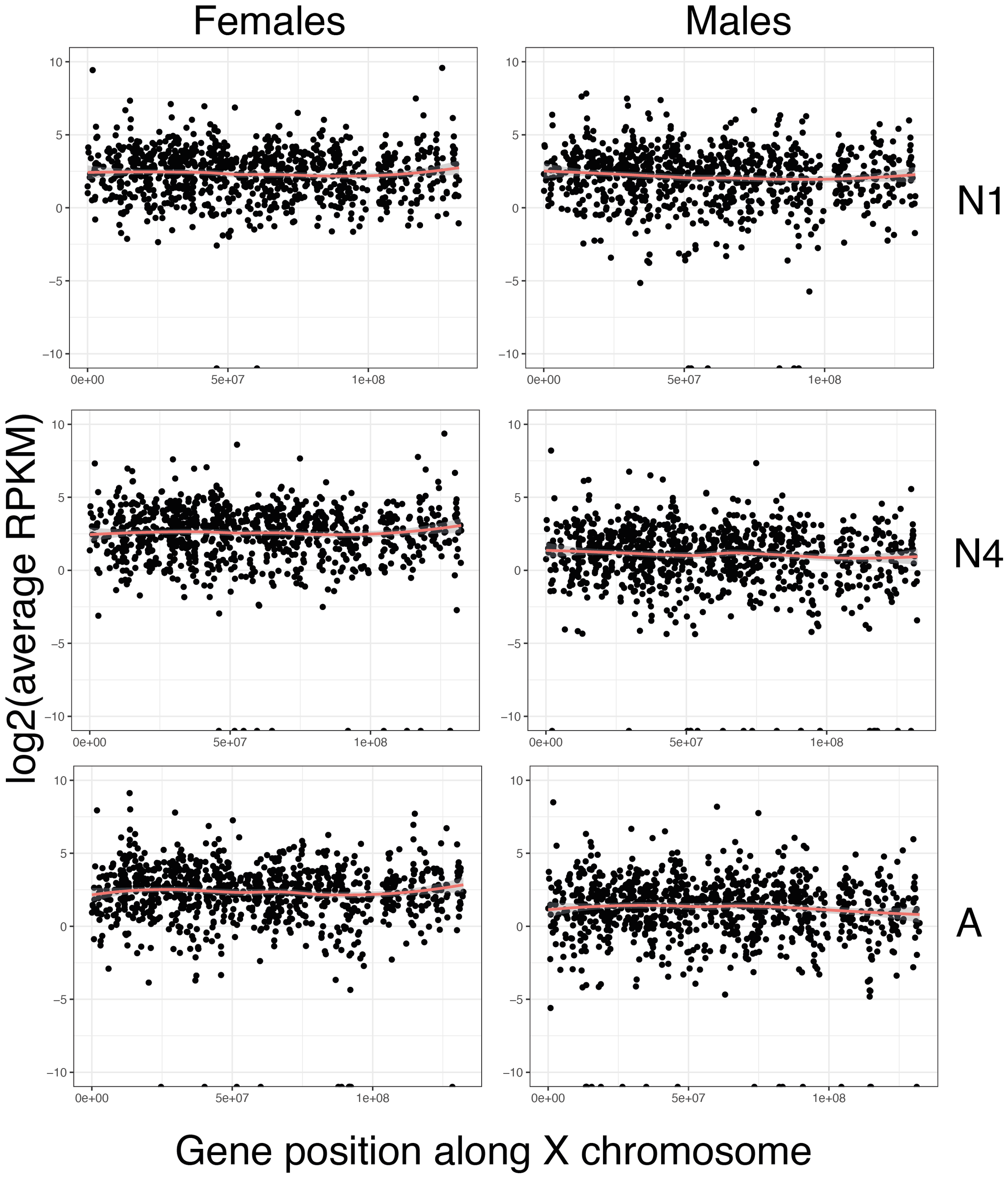

Supplement: S7 Fig — The red line in each panel shows the loess smoothed curve. (TIF) [file pgen.1011615.s012.tif]

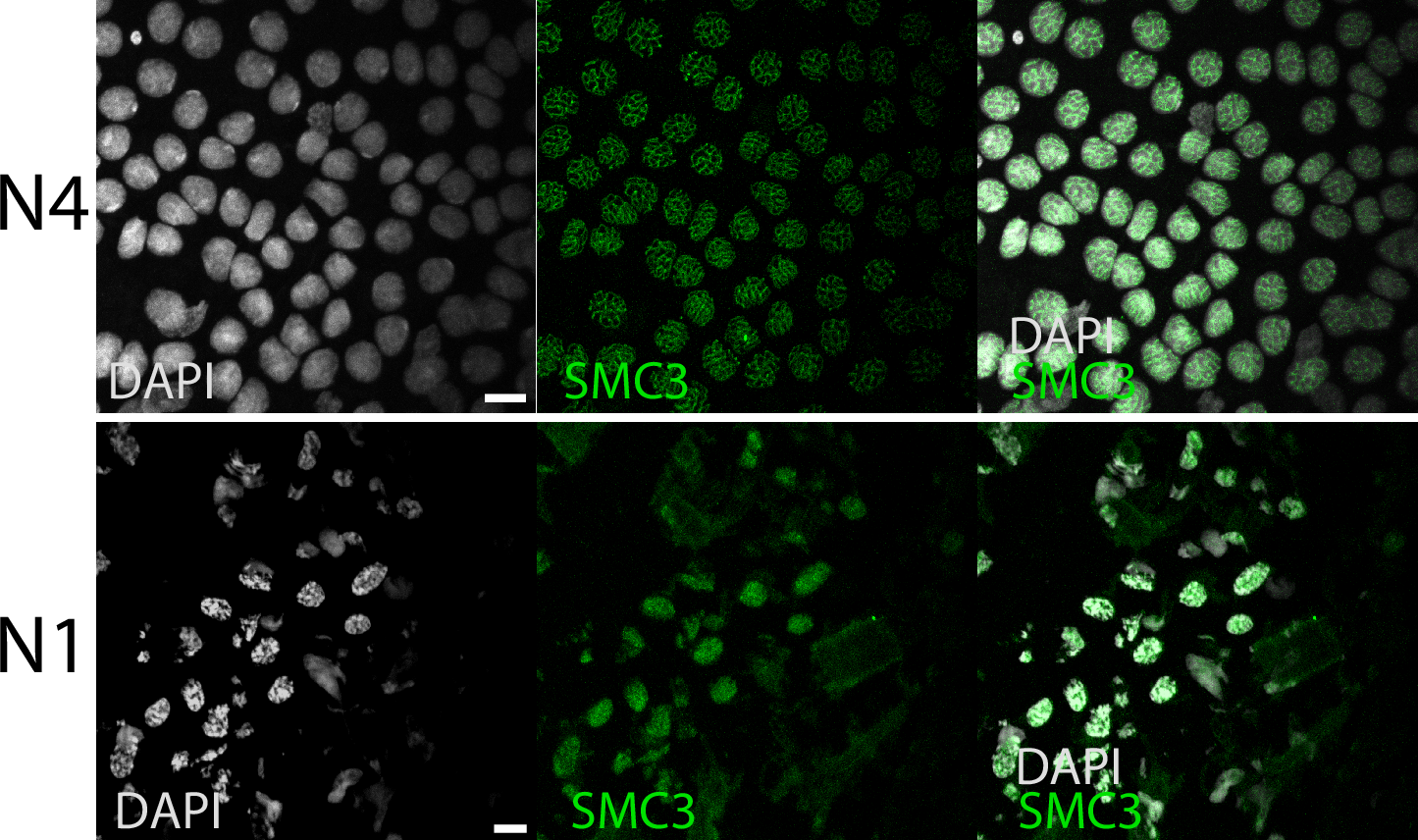

Supplement: S8 Fig — SMC3 is a protein of the cohesin complex marking chromosome axes during meiosis 1. No cells in meiosis I were detected at N1 (scale bar 10µm), while numerous cells at this stage were detected at N4 (scale bar 20µm). (TIF) [file pgen.1011615.s013.tif]
