# Supplementary material for: Dynamics of X chromosome hyper-expression and inactivation in male tissues during stick insect development
Source: PLoS Genet. 2025 Mar 10;21(3):e1011615. doi: 10.1371/journal.pgen.1011615 (PMC11957559; doi:10.1371/journal.pgen.1011615)
Supplement: Attachment — Submitted filename: Response.docx [file pgen.1011615.s014.docx]

Dear PLoS Genetics Editors,

Please find attached the revised version of our manuscript entitled “'Dynamics of X chromosome hyper-expression and inactivation in male tissues during stick insect development'” for consideration of publication in PLoS Genetics.

We thank the three reviewers for their constructive suggestions which helped us improve our manuscript, as well as recognizing the novel and important contributions of our study in a non-model organism. The major point raised by the handling editor (and reviewer#1) is that the GitHub repository for the code(https://github.com/JelisavetaDjordjevic/Dosage_compensation) and the NCBI SRA accession (PRJNA1126215) for the sequence data need to be available. This is now the case. We also uploaded our figure files to the PACE digital diagnostic. Finally, we addressed all the points raised the reviewers, which represent mostly requests for clarification, as detailed in our point-by-point replies below. We hope that the applied changes meet with your approval.

Kind regards,

for the authors

Jelisaveta Djordjevic

Reviewer #1: The manuscript by Djordjevic, Schwander and colleagues provides a timely characterization of X chromosome-associated gene expression changes in the stick insect Timema poppensis. To do this, the authors create a new chromosome-level assembly, followed by an analysis of sex-specific gene expression across developmental stages and tissues with the finding that dosage compensation is complete across tissues and stages. The authors then investigate the male germline more specifically and discover the presence of meiotic sex chromosome inactivation. This latter part is conclusive based on 1) the fact that repression goes way beyond the 2-fold difference expected solely due to the lack of dosage compensation (authors asses it by RNA-seq) 2) ChIP-seq of H3K9me3 showing enrichment of this repressive mark in male germline and 3) immunostainings in germ cells showing DAPI dense and Pol2-depleted nuclear areas.

Although there is no cytological proof that these dense areas are the X, the combination with ChIP-seq make it a well-supported evidence for MSCI occurring in Timema. The latter part is especially interesting because the male Drosophila germline does not exhibit MSCI, raising questions of how widespread this mechanism is and how quickly it evolves.

A highlight of the manuscript is its comprehensive sampling of transcriptome data across stages and tissues. While the manuscript itself focuses on dosage compensation and MSCI, this dataset will be certainly of great interest for a broad audience (for example, it could be used to study the evolution of gene expression across stages in different insects, or between sexes or between tissues). Meaning the dataset on its own is excellent and deserves publication in a journal like PLOS Genetics! I would also like to compliment the authors for their efforts in having a concise story with a clear focus on dosage compensation and thus, having “digestible” main figure panels. Another strength is that the authors attempt to complement their genomics analyses with cell biological work, which is not trivial in non-models such as Timema - protocols and antibodies are often unavailable / not easily adaptable.

I don’t think the paper has any experimental weaknesses but there are a couple of instances where there is a lack of clarity in data representation and/or writing and/or lack of context.

We thank this reviewer for their positive evaluation of our work and constructive suggestions listed below, which we now all included in our manuscript. (And apologies for the lack of line numbers in the previous version, we have now added line numbers for simplified reference to manuscript sections).

Here are some suggestions and comments that I think will make the paper more helpful for non-specialists in the field (unfortunately there were no line numbers in the manuscript, I hope it is clear to what I am referring to).

- Text: P.3/4 Introduction – There is some debate on whether mammalian X inactivation is a form of “dosage compensation”. I find this in the current intro text confusing considering that the second part of the manuscript deals with meiotic sex chromosome inactivation in males (but XCI in mammals occurs in females, while mammalian males have MSCI – the silencing of X-genes in the male germline thus a priori cannot occur due to a lack of the canonical X inactivation process which happens in females). Furthermore, C.elegans is a hermaphrodite and we generally have no idea how widespread the two-fold dampening is across taxa, because it has not been found elsewhere (in Danaus plexipus there is some suggestion on dampening, but not via H3K4me). These facts per se are interesting for our field, but I don’t think they need to be covered in the intro for this paper (besides that dosage compensation mechanisms are anyhow not the topic of the current study). I would recommend to focus the dosage compensation part in the intro on diversity in insects, maybe perhaps briefly stating mechanisms are different and mentioning that a current “hot” theme in the field is to investigate whether this process occurs in all tissues/dev stages. This would give a bit more space also to the discussion of ancient (non-dipteran insects) vs. different muller element becoming X in dipterans (the part about “derived” sex chromosomes later on this page is not well explained). Later when authors discuss the mosquito work, it sounds a bit like dosage compensation is regulated via alternative splicing the way the sentence is phrased. Alternative splicing regulates the sex-specific expression of SOA/007 protein in only males, but whether SOA/007 itself is a splicing regulator has not been investigated to date. The section at the beginning of p.4 is also not really clear, since male mammals have MSCI: X inactivation and its components are present in females, obviously this cannot explain why the X is repressed in the mammalian male testis (in males there is no Xist). In other words – I would recommend rewriting the introduction and focusing first on DC in insects (dipterans and non-dipterans) and MSCI. The presence of MSCI in insects as proven here is in my opinion also the most novel finding – besides the value of the dataset on its own (as stated above).

We have removed the part about the detailed molecular mechanisms as suggested by this reviewer. However, we kept the description of broad differences among non-insect species for how dosage compensation between the sexes is achieved (i.e., via upregulation of the X in males, and/or downregulation of both Xs, inactivation of one X in females). Since we document upregulation of the single X in males, and our data suggest that the expression from both Xs may be dampened in stick insect females, we prefer keeping a broad description in the introduction as well.

- Text, p.14 - “However, the X chromosome in stick insects has been conserved for at least 120 Mya [13, 53] and shares most of its content with the X chromosome in other insect orders that diverged over 450 million years ago [15], making it impossible to infer ancestral transcription levels.”

o Muller F in Diptera has reverted back to an autosome, so probably not meaningful for comparison. But what about other insects such as springtails? Or other insects (termites?) (see also Toups et al. paper)? I don’t mean that the authors need to do this analysis, but I would find it helpful to add this consideration in the text instead of giving such an absolute statement.

We now reformulated the relevant statements in the introduction (see lines 77-81) and results (lines: 359-364) for clarification. The key point we want to make is that because of the very deep conservation of the X chromosome in stick insects (and beyond), the inference of expression levels of the autosomal ancestor of the current X (i.e., before the autosome acquired a sex-determining function) is challenging. We therefore cannot know whether the reduced X-linked expression (relative to expression on autosomes) in females was already a feature of the autosomal X precursor or emerged later.

- Text, p.13.-14. I found the intermingling of discussion and results not optimally solved in this area of the manuscript. Perhaps this could be streamlined a bit better (better solved in later Figures/paragraphs).

We clarified several parts in this section (see also replies to previous comments, which referred to some sections now on pages 16-17).

- Text.p.20 – “The presence of MSCI in stick insects as we document here and lack thereof in D. melanogaster supports the first hypothesis. A key difference between male meiosis in Timema stick insects and D. melanogaster is that meiosis is achiasmatic in …” Very nice finding – the authors could stress that part even more (perhaps even in abstract) since they are of very general interest for the meiosis and DNA repair field.

We streamlined this section in the discussion to give it more weight. We are excited about the possible link with achiasmy (which we are currently investigating further) and are happy to read that this reviewer agrees with our interpretation. However, with a single contrast (*Drosophila* vs *Timema*) we decided that the link with achiasmy is too speculative to be included in the abstract.

Figures:

- Fig. 1 – Since stick insect development, the nymph-stages of hemimetabolous insects as well as germline development (meiosis at which stages?) are likely not a concept familiar to each reader – could the paper (Fig. 1A) start with an overview / schematic of the stages/tissues studied here, and key developmental transitions in the development of the germline (no meiosis / meiosis)?

We have now added such a figure (Page 14, Figure 1A).

- Fig. 1 – The y-axis scale is not helpful – one cannot really see the median line well (which is what matters here). Can the y-axis be separately scaled for each of the plots? Perhaps the authors can also provide density plots as in 3C (for example for the brain / antenna in adults, where there seems to be an interesting exception)? (along the same lines text, p. 14 “While dosage compensation is complete between males and females (Figure 1A), X-linked genes generally have significantly lower expression than autosomal genes in male somatic tissues (Figure 1B; Supplemental Table S3).” This cannot be really seen in the Figure. In my opinion the whiskers could be even removed, keeping simply the box.

For improved readability and clarity, we have adjusted Figure 1 (and others) by replacing the dark blue with a lighter shade and added notches to the boxes to better emphasize the median values. To further improve readability, we reduced the y-axis limits and included significance markers directly on the plots. However, we kept the Y-axis scale uniform across plots to facilitate comparison across figures. Additionally, we have added density plots, now provided in the Supplement, to further illustrate patterns for adult brain and antenna tissues, where notable exceptions are observed.

- Fig. 2 – Interesting that the X shows more tissue-specific genes. Any preference for certain tissues here? I would find a bit more extra presentation on those results besides Tau index worthwhile.

Our analyses do not focus on identifying individual tissue-specific genes on the X chromosome. Instead, our results demonstrate that genes on the X chromosome exhibit overall more tissue-specific expression patterns compared to autosomal genes, as measured by the Tau index. The latter is relevant for evaluating global limits to X regulation, which is the important point for the purpose of this study (we are currently investigating gene-specific expression pattern of X-linked genes in a different context). We made sure all the relevant text sections are clear with respect to this point.

- Fig. 4 – Immunofluorescence (IF): Which phosphorylation state of Pol2 does the antibody detect? Pan Phospho Pol2? Ser2P? Ser5P? Can this be clarified and also amended in the main Figure label in the IF? Also in the text on p.17 “It also lacks a RNApol-II signal, a marker for transcriptional activity, in otherwise transcriptionally active cells (Figure 4).” – please clarify accordingly.

The antibody used detects Pan Phospho Pol2. We have clarified this in both the figure legend and the main text as recommended.

- Fig. 5 – IF: From the methods it seems you have a double labelling for also SMC3 – can this data also be provided?

We did not perform double staining for H3K9, as this is currently not feasible: the H3K9me3 and SMC3 antibodies which work well in stick insects are derived from the same host species and therefore cannot be co-stained. We have now clarified this in the Methods section (lines: 256-264).

- Fig. 4/5 – can the style be unified? Some panels have no scale bar, sometimes a single scale bar without label of um, sometimes with label of um. Font size different, different arrow styles. Once magenta as color, once red. DAPI in blue, once in white. The overview Figures (B panel in Fig. 5) is missing in Figure 4 – this should be provided (especially for p-Pol2) – how often do you see the “hole” (I think I see it for DAPI in Fig. S8 - btw, here the labels are outside the figure, not inside, and scale bar is yet another style). A bit more effort on the consistent data representation in the IF would pay off.

We have revised the figures for consistency as suggested. All panels now include scale bars with uniform labeling (including µm where applicable), standardized font sizes, and consistent arrow styles. We have also aligned the color schemes, with DAPI consistently in white and red replacing magenta where appropriate. Additionally, we included an overview panel in Figure 4 (similar to the B panel in Figure 5) for p-Pol2 to enhance clarity. We have adjusted figure labels in Figure S8 to be inside the panels with consistent scale bar formatting.

- Fig. 5 – ChIP-seq

o Legend: “Smoothing lines (method “gam”) for each scaffold are in white.” – Can you please explain what this means in methods?

We have now explained this part in the methods.

o What does the increase of signal towards the chromosome end mean? Is that subtelomeric heterochromatin? Can you pls explain?

Yes, the signal increase towards chromosome ends most likely reflects subtelomeric heterochromatin as well as pericentromeric regions (several chromosomes are telocentric). However, we here focus solely on between chromosome variation as intra-chromosomal variation falls outside the scope of the current paper (and would require different types of analyses). We therefore prefer not discussing this variation here.

o Can the x-axis be relabeled to the chromosomes and not as a single line? Why is the X blue? It was orange before.

The x-axis represents cumulative base pairs across chromosomes for the entire genome (which is a standard display used in genomic studies). For example, if Chromosome 1 is 1000 bp, Chromosome 2 is 500 bp, and Chromosome 3 is 300 bp, then the x-axis spans 1800 bp, with Chromosome 2 starting from 1001 bp, and so on. To further clarify, we have added labels above the X-axis to indicate chromosome numbers.

Regarding the color scheme, the X chromosome is consistently shown in blue for males, red for females, and orange when displaying male-to-female ratios.

Supplementary Information:

- Supp Figure S1 – what is Tps? What does the x-axis represent? Aren’t those the scaffolds from 1-12? Why representing this as one single axis in bp (the chromosomes have very different sizes, aren’t they?)

“Tps” stands for *Timema poppense*, and we have now removed this label from the figure and clarified the legend. The x-axis represents cumulative genomic position. Yes, the chromosomes differ in size, which is why they appear smaller towards the end of the x-axis, given that chromosomes are ordered by size, from largest to smallest.

- Supplemental Table S3 – I appreciate the intention for full completeness of all tests performed – could the authors think of a way to include the significant comparisons also in the main figure?

We appreciate the reviewer's suggestion for enhancing clarity. We have now added significance labels to the main figures to highlight the significant comparisons alongside the full set of tests provided in Supplemental Table S3.

- Supplementary Figure S1/ Legend – Can you explain what this means “The line in each panel represents a loess smoothed curve.”

We have added an explanation to the figure legend.

- Supp. Figure S2 – is it bp?

Yes, it is. We have now clarified in figure legend.

- Supplemental Figure S3. / The authors have produced a beautiful chromosome-level assembly. Why keeping the nomenclature of scaffolds? I would recommend using chr1-chr12, and saying chr3 (X chromosome).

We appreciate the positive feedback on our chromosome-level assembly. We have updated the legend as suggested, replacing the nomenclature of scaffolds with chr1-chr12 and specifying chr3 as the X chromosome.

- Supp. Figure S3/S4 – y-axis are not ideally chosen (I guess it’s what comes out of R/ggplot2 by default) – however one can hardly see the differences / boxplots. I would manually adjust for each tissue so that the whiskers fit optimally in (e.g. ±2 for the Antenna in Fig. S3). Fig. S4 it’s impossible to see the median line. I find it well done / visible in S6A as a general guidance.

We have decreased the Y-limits, but we would like to keep the same Y axis across plots for comparability between the tissues (see also our reply to a similar comment above on the figures of the main text).

- Not sure how helpful the Supplementary Tables S2,S3,S5 are – they go across many pages making it really difficult to get an overview. Can one make a Supp. Figure instead and represent the raw values perhaps on another repository (github?)?

Yes, these tables are now provided as separate Supplementary Tables.

Methods / General:

- H3K9me3 ChIP-seq – can you provide a bit of QC please? Maybe I missed it but where is the info on replicates, number of mapped reads (%), where the enrichment is found (peak calling?)? Any other insights, except for the X?

Our focus is on detecting overall signal differences between the X chromosome and autosomes, so we have concentrated on assessing broad differences (based on a pool of tissues from over 40 individuals) rather than fine-scale (intra-chromosomal) patterns of variation. For quality control, we have provided information on replicates and mapped read counts (including percentages), which are now included in methods, page 12-13, lines: 315-323.

- Generally letter panels (A, B, C) come in very different font styles (size, bold/not bold) – can you homogenize the layout? Also legends could sometimes profit from bigger font size.

Improved as suggested.

- Methods: “Immunolabelling of X chromosomes” should be rephrased to “Immunfluorescence staining” (because you are not labelling the X chromosome here). Same paragraph, can you also give more details on the microscopy (magnifications, objectives, how you processed the images (Fiji?), do we see single planes or Max-intensity projections? How were they contrasted). How many nuclei / individuals / pictures were taken?

We rephrased to “Immunofluorescence staining”. We have added the details regarding the microscopy in methods (page 11, lines: 280-281) or figure legend (Figure 4).

- For the dosage compensation analysis (methods, p.9): I did not quite understand what exactly has been done in edgeR and how the averages between males and females were calculated. How were for example replicates taken into account? How many genes did you compare versus each other? Is this the information provided in Supplemental Table S4? I think it would be nice to have this provided in methods as well as it looks a bit hidden / not easy to grasp at the moment. Can the authors provide all EdgeR results as supplementary Excel files (like what are the genes differentially expressed between males and females in the different stages, what are those, etc.)?

We used EdgeR to normalize expression depending on the library sizes, for between samples comparability. We also used EdgeR to exclude genes with low expression. (We now provide the number of genes used in the different analyses in the methods, [page 9-10, lines 239-241]). We further calculated RPKM values, which allows us to compare genes with different lengths within sample. We then calculated average RPKM for each sex (mean) for number of replicates.

Supplemental table 4 just shows the number of Sex-biased genes in gonads, as well as the total number of expressed genes in each developmental stage within gonads. For the purpose of this paper we just needed quantification of differentiation in gonads, independently of putative functional annotations. We also provide a table with the specific counts on the git hub, as well as the complete code. Note that the functional annotations and quantification of sex-biased genes in different tissues are the focus of a different study and are therefore not provided or discussed here.

- I went to the github repository but was unable to find the code (neither through the link nor by going via the first author’s name).

The github repository is now publicly open.

- The Raw Sequencing Data could not be found on NCBI SRA with the accessions provided.

The embargo is now lifted.

I apologize for the lengthy peer-review, but I think most of these issues are small, but relevant details that can be quickly and easily fixed by the authors.

These are all useful comments, and we thank the reviewer for taking the time to carefully go through our manuscript and writing them down!

Reviewer #2: Djordjevic et al. investigate dosage compensation (DC) in the stick insect Timema poppense, a species with X0 sex determination. They assemble a new genome sequence with long (ONP) and Hi-C reads, using the coverage difference between females and males to identify the X chromosome. By comparing RNA-seq data from both sexes, as well as from multiple tissues and developmental stages, they show that there is complete dosage compensation of the X chromosome in the somatic tissues of males. In male reproductive tissues of the 4th nymphal stage or adults there is no evidence of DC and average X-linked gene expression is reduced more than two-fold, suggesting that the X is actively silenced. This is supported by immunolabeling of male gonad tissue, which shows an absence of X transcription (RNA Pol2) and an enrichment of silencing histone modifications (H3K9me3). These results are consistent with the occurrence of MSCI (meiotic sex chromosome inactivation) in Timema.

The authors have done a thorough job investigating DC in a stick insect. It is especially notable that they have examined expression in multiple tissues and developmental stages and that they have performed immunohistochemistry to test for epigenetic marks associated with chromatin silencing. Below are a few suggestions that I think would improve the manuscript.

1) Abstract: "Stick insects are characterized by XX/XO sex determination and an X chromosome which likely evolved prior to the diversification of insects over 450 Mya" - would be better as "Stick insects are characterized by XX/XO sex determination, with an X chromosome that likely evolved prior to the diversification of insects over 450 Mya"

Changed as suggested.

2) Page 12: "unambiguously identify the third largest scaffold as the X chromosome" - it would be helpful to mention how large this scaffold is and what proportion of genes in the genome are X-linked. It sounds like the X represents a larger proportion of the genome in Timema than in mammals or (maybe) Drosophila. The size of the X may play a role in the evolution of DC as the larger the X the more likely there are to be haploinsufficient genes that need to be compensated. Perhaps this contributes to differences between taxa?

We have added size and proportion of genes on the X, page 13, line 317.

Indeed, it would be very interesting to test whether the size of X contributes to differences between taxa. However, it would be necessary to compare across a large number of species with variable % of X-linked genes for conclusive results. Briefly both *Timema* and mammals have around ~6% of genes on the X.

3) The authors should indicate significant differences between X and A with asterisks (or "ns" if not significant) on figures 1, 2, and 3.

Significance signs are now indicated.

4) Some authors distinguish between dosage compensation (equalizing expression of X and autosomes in males) and dosage balance (equalizing expression of X between males and females). For example, see Gu et al., https://doi.org/10.1016/j.cub.2019.09.056. Since the authors test for both of these, it might be helpful to implement this terminology in their manuscript.

Yes, we have now mentioned “dosage balance” in our results, page 16, lines 350-351. However, since we are unable to compare X to its ancestral levels of expression, we cannot fully apply this terminology.

5) Page 14: To explain their observation that the X generally has lower expression than the autosomes, the authors state that "a limit to the maximal transcriptional activity of a haploid chromosome is notably believed to explain X chromosome gene content and reduced expression levels in therian mammals and Drosophila fruit flies". They then go on to support this hypothesis by noting that the X has higher tissue specificity than the autosomes in Timema and mammals, and that broadly expressed genes tend to have higher expression. I think there are a couple problems with this argument. First, in Drosophila the opposite pattern is observed: X-linked genes are less tissue specific than autosomal genes (Mikhaylova and Nurminsky, https://doi.org/10.1186/1741-7007-9-29; Meisel et al., https://doi.org/10.1101/gr.132100.111).

Second, since dosage compensation occurs at the cellular level, it is not the total amount of expression in the organism that is relevant, but instead the amount per cell. Many tissue-specific genes have very high expression in cells of a particular tissue, but their overall expression level may be low when averaged over the entire organism.

We now added that what we observe is similar to mammals but opposite to *Drosophila.*

Indeed, gene expression levels can differ depending on the cell type. We have now also added a sentence about composite tissue and averaging effect across cell types, page17, lines:374-377.

6) Bottom of page 14: "Lastly, X chromosome degradation could also contribute to lower expression" - I'm not sure the paragraph needs to begin with "Lastly", as the authors don't really present a list of explanations in the preceding paragraph.

We changed from “Lastly” to “Additionally”, line 378.

7) Bottom of page 17: I think the authors oversimplify the discussion regarding the presence/absence of MSCI in Drosophila. For example, studies that used reporter genes or transposed endogenous genes in which gene dose could be controlled showed clear evidence for suppression, but not complete inactivation, of X-linked gene expression in the male gremline (Hense et al., https://doi.org/10.1371/journal.pbio.0050273; Kemkemer et al., https://doi.org/10.1038/hdy.2013.86; Landeen et al., https://doi.org/10.1371/journal.pbio.1002499). scRNA-seq studies cannot control gene dose and, thus, cannot distinguish between the effects of lack of DC and those of MSCI in many cases. For example, ref. 51 cited in the manuscript seems to favor an X-inactivation model based on observations of the X and fourth chromosome, while the authors of ref. 52 explicitly state that they cannot make a conclusion about MSCI. The other cited references focus more on the mechanism to draw a conclusion, such as the absence of H3K9 methylation (ref. 64).

In the end, I think the authors conclusion mostly holds: in Timema, similar to mammals, there appears to be complete MSCI. The situation is different in Drosophila, where there does not appear to be complete MSCI, but rather partial suppression of X chromosomal gene expression in the male germline, which occurs through an unknown mechanism that does not involve H3K9 methylation. I suggest they alter their wording to reflect this.

We have reformulated this section to provide a more nuanced presentation of X expression in meiotic cells of *Drosophila* males. The main point we want to make is that reduced expression of the X in *Drosophila* germ cells is not achieved via a mechanism similar to MSCI in mammals, see page 20, lines 429-433.

Reviewer #3: Overall evaluation

This work addresses a model epigenetic process, dosage compensation, in non-model organism. The choice of an organism presumed to have an ancestral karyotype, and thus likely to retain an ancient mode of compensation, is particularly exciting. The authors have done a great deal of careful work, but there are logical lapses in presentation, and omission of critical details, that detract from impact and reader confidence.

Major points:

1) The authors assume that the mode of dosage compensation in T. poppense is increased expression from the male X chromosome, but this has not been demonstrated. Have the authors eliminated the possibility that both female X chromosomes decrease in expression? This is not a minor point. Even in the well-studied fly and mammalian systems the precise mechanism of achieving compensation is still being debated.

Figure 1b demonstrates that 1X has equal expression to female 2Xs as well as similar levels to autosomes, meaning that 1 X has to double the expression, hence the upregulation of the X in males. However, we do agree that additional dampening of X expression in females is possible or perhaps even likely, and we now clarify these points in the results section (page 16, lines: 361-363).

2) In A. gambiae, DC is regulated via sex-specific alternative splicing of the SOA gene, and activation of the male X occurs, although it does not involve H4K16ac. On page 3 it would be more appropriate to say that activation by SOA is poorly understood rather than unknown.

We have removed the details regarding the molecular mechanisms in insects following a suggestion of reviewer#1.

3) The authors do not confirm identity of the X in Fig. 4. As far as can be told from this manuscript the assumption is made that the condensed, DAPI-bright spot must be the X. I realize that confirmation may be beyond their expertise, but, if so, indicate that identity is presumed.

Our complementary ChIP-seq data clearly shows enrichment of H3K9me3 marks on the X and not on any other chromosome (Figure 5c); combining the data from our different approaches (figure 4 and 5c), we can therefore conclude that X-inactivation involves silencing histone marks, and that the H3K9me3 territory (overlapping with the DAPI-bright spot) in fact does correspond to the X. We have clarified the text in our manuscript accordingly to avoid confusion, page 11, lines: 283-285.

4) Indicate how many individuals/nuclei contributed to the histological identification of MSCI. How frequently was the SC and a DAPI-bright spot detected, and is Fig. 4 a representative image?

We have now added additional zoomed out image to the main figure 4 where we show many cells with inactivated X. Yes, Figure 4 is a representative image, but we have examined slides from up to 10 individuals and documented similar patterns across many individuals from different *Timema* species in a follow-up study (https://doi.org/10.1101/2024.09.05.611401).

5) The authors claim that X-linked genes are expressed at lower levels in somatic tissues, but this is certainly not apparent in figure 1B. Table S3 is presented in support, but only contains p values. I am confident that the authors know their stuff but what is presented here does not enable the reader to reconstruct their argument. As a side note, low expression of X-linked genes in both sexes supports the idea that dosage compensation might occur through repression of both female X chromosomes – or by a combination of activation in males and repression in females. Can the authors eliminate these possibilities?

We have now added significance signs to the main figure, for easier interpretation. Statistical results are separately presented in excel files.

6) The onset of MSCI is nicely demonstrated by gene expression and quite convincing. But the legend to Fig. 3 states that compensation is absent in the late germ line, implying developmentally controlled dosage compensation. As the authors ultimately conclude that that reduced expression in male gonad in N4 is due to MSCI, a more nuanced figure legend would be appropriate.

We have revised the figure legend to provide a more nuanced title.

7) Indicate number of replicates used for RNA sequencing.

Number of replicates are indicated in Supplemental Table 1. We have added further information in the legend to clarify that numbers correspond to RNA-seq samples.

Minor points

1) What stage are the germ cells in Fig. 5 A and B?

Pachytene stage, now clarified in the legend.

2) Edit manuscript for English usage and clarity throughout.

Done

3) Discussion of chiasmatic vs. achiasmatic meiotic risk (bottom p. 20) hard to follow.

This section was improved and clarified, page 22, lines 446-464.

4) The blue fill in figures 1B, 2, 3B, S4, S5 and S6 is so dark that the median disappears.

Dark blue is now replaced by lighter blue color.

Have all data underlying the figures and results presented in the manuscript been provided?

Large-scale datasets should be made available via a public repository as described in the PLOS Genetics data availability policy, and numerical data that underlies graphs or summary statistics should be provided in spreadsheet form as supporting information.

Reviewer #1: No: Github codes and NCBI data could not be found.

Reviewer #2: Yes

Reviewer #3: Yes

PLOS authors have the option to publish the peer review history of their article (what does this mean?). If published, this will include your full peer review and any attached files.

If you choose “no”, your identity will remain anonymous but your review may still be made public.

Do you want your identity to be public for this peer review? For information about this choice, including consent withdrawal, please see our Privacy Policy.

Reviewer #1: No

Reviewer #2: No

Reviewer #3: No
